# Supplementary material for: Super-resolving microscopy reveals the localizations and movement dynamics of stressosome proteins in Listeria monocytogenes
Source: Commun Biol. 2023 Jan 14;6:51. doi: 10.1038/s42003-023-04423-y (PMC9840623; doi:10.1038/s42003-023-04423-y)
Supplement: Supplementary file 2 — Supplementary Information [file 42003_2023_4423_MOESM2_ESM.pdf]

Supplementary Information

Super-resolving microscopy reveals the localizations and movement dynamics of stressosome proteins in *Listeria monocytogenes*

Buu Minh Tran[1], Dmitrii Sergeevich Linnik[1], Christiaan Michiel Punter[1], Wojciech Mikołaj Śmigiel[1], Luca Mantovanelli[1], Aditya Iyer[1], Conor O'Byrne[2], Tjakko Abee[3], Jörgen Johansson[4] and Bert Poolman[1]

[1] *Department of Biochemistry, University of Groningen, Groningen, the Netherlands*
[2] *Microbiology, School of Biological & Chemical Sciences, National University of Ireland Galway, Galway, Ireland*
[3] *Laboratory of Food Microbiology, Wageningen University & Research, Wageningen, the Netherlands*
[4] *Department of Molecular Biology, Umeå University, Umeå, Sweden*

Keywords: *Listeria monocytogenes,* protein localization, protein diffusion, Prli42, RsbR1, RsbL, stressosome, super-resolution microscopy, single-particle tracking, single-molecule displacement mapping

Table 1. Molecular weight (M_W), pI, and net charge of proteins of interest

Protein/Fusion	Theoretical M_W (kDa)	Average_pI	Average net charge at pH 7.5
mEos3.2	26.2	7.33	-0.49
mEos3.2-prli42	30.1	8.14	1.49
mEos3.2-RsbR1	57.8	5.50	-14.53
mEos3.2-RsbL	55.2	5.29	-21.46
RsbR1	31.6	4.70	-14.28
RsbL	28.8	4.60	-20.21

Protein sequences

>RsbR1 (genome) || Molecular weight: 31620.5 Da

MYKDFANFIRTNKADLLNDWMNEMEKQSDQLINDIAKEAMYEETSKEFVDLIVSNVTENGS
KFNEKLDDFAEKVVHLGWPIHFVTTGLRVFGLLVYTAMRDEDLFLKREEKPEDDAYYRFET
WLSSMYNKVVTAYADTWEKTVSIQKSALQELSAPLLPIFEKISVMPLIGTIDTERAKLIIENLLI
GVVKNRSEVVLIDITGVPVVDTMVAHHIIQASEAVRLVGCQAMLVGIRPEIAQTIVNLGIELD
QIITTNTMKKGMERALALTNREIVEKEG

>RsbL (genome) || Molecular weight: 28816.77 Da

MTAYPQFDVILKALNLSSVGVIITDPEQKDNPIIFVNTGFENITGYAKEEALGSNCHFLQGDDT
DKEEVAKIRHAINEKSTANVLLKNYRKDGTSFMNELTIEPIYDDHEHLYFVGIQKDVTTEHD
YQLELEKSLTEIEKLSTPIVPIKENICVLPLIGSLTHDRFQHMSEYVSEYMDHGKEDYLIMDLS
GLAEFNEDAVMNLVKFHGFMKLTGVELIITGISPKFAMTLIRYEENLASLTTYSTIKEALQFY

>mEos3.2 (in pNF mEos3.2) || Molecular weight: 26217.64 Da

MGSAIKPDMKIKLRMEGNVNGHHFVIDGDGTGKPFEGKQSMDLEVKEGGPLPFAFDILTTAF
HYGNRVFAKYPDNIQDYFKQSFPKGYSWERSLTFEDGGICNARNDITMEGDTFYNKVRFYG
TNFPANGPVMQKKTLKWEPSTEKMYVRDGVLTGDIEMALLLEGNAHYRCDFRTTYKAKEK
GVKLPGAHFVDHCIEILSHDKDYNKVKLYEHAVAHSGLPDNARR**GGTGGS**

>mEos3.2-Prli42 (in pNF mEos3.2-prli42) || Molecular weight: 30098.58 Da

MGSAIKPDMKIKLRMEGNVNGHHFVIDGDGTGKPFEGKQSMDLEVKEGGPLPFAFDILTTAF
HYGNRVFAKYPDNIQDYFKQSFPKGYSWERSLTFEDGGICNARNDITMEGDTFYNKVRFYG
TNFPANGPVMQKKTLKWEPSTEKMYVRDGVLTGDIEMALLLEGNAHYRCDFRTTYKAKEK
GVKLPGAHFVDHCIEILSHDKDYNKVKLYEHAVAHSGLPDNARR**GGTGGS**PWMTNKKVVR
VVVILMLIAIVLSSVLTGVLMFLVD

>mEos3.2-RsbR1 (in pNF mEos3.2-RsbR1) || Molecular weight: 57833.06 Da

MGSAIKPDMKIKLRMEGNVNGHHFVIDGDGTGKPFEGKQSMDLEVKEGGPLPFAFDILTTAFHYGNRVFAKYPDNIQDYFKQSFPKGYSWERSLTFEDGGICNARNDITMEGDTFYNKVRFYGTNFPANGPVMQKKTLKWEPSTEKMYVRDGVLTGDIEMALLLEGNAHYRCDFRTTYKAKEKGVKLPGAHFVDHCIEILSHDKDYNKVKLYEHAVAHSGLPDNARR**GGTGGS**GSYKDFANFIRTNKADLLNDWMNEMEKQSDQLINDIAKEAMYEETSKEFVDLIVSNVTENGSKFNEKLDDFAEKVVHLGWPIHFVTTGLRVFGLLVYTAMRDEDLFLKREEKPEDDAYYRFETWLSSMYNKVVTAYADTWEKTVSIQKSALQELSAPLLPIFEKISVMPLIGTIDTERAKLIIENLLIGVVKNRSEVVLIDITGVPVVDTMVAHHIIQASEAVRLVGCQAMLVGIRPEIAQTIVNLGIELDQIITTNTMKKGMERALALTNREIVEKEG

>mEos3.2-RsbL (in pNF mEos3.2-RsbL) || Molecular weight: 55161.5 Da

MGSAIKPDMKIKLRMEGNVNGHHFVIDGDGTGKPFEGKQSMDLEVKEGGPLPFAFDILTTAFHYGNRVFAKYPDNIQDYFKQSFPKGYSWERSLTFEDGGICNARNDITMEGDTFYNKVRFYGTNFPANGPVMQKKTLKWEPSTEKMYVRDGVLTGDIEMALLLEGNAHYRCDFRTTYKAKEKGVKLPGAHFVDHCIEILSHDKDYNKVKLYEHAVAHSGLPDNARR**GGTGGS**EFTAYPQFDVILKALNLSSVGVIITDPEQKDNPIIFVNTGFENITGYAKEEALGSNCHFLQGDDTDKEEVAKIRHAINEKSTANVLLKNYRKDGTSFMNELTIEPIYDDHEHLYFVGIQKDVTTEHDYQLELEKSLTEIEKLSTPIVPIKENICVLPLIGSLTHDRFQHMSEYVSEYMDHGKEDYLIMDLSGLAEFNEDAVMNLVKFHGFMKLTGVELIITGISPKFAMTLIRYEENLASLTTYSTIKEALQFY

RsbR1: Blue sequence/ RsbL: Dark golden sequence/ mEos3.2: Green sequence/ Prli42: Magenta sequence/ **GGTGGS:** hexa-amino acid linker

Table 2. List of strains and plasmids used in this paper

Bacteria strains	Reference/source
Escherichia coli	
K12	NovaBlue
MC1061 *ΔarcB* (Erythromycin sensitive)	[1]
Listeria monocytogenes	[2]
EGD-e	K. Boor (Connor O'Byrne's lab)
EGD-e *ΔrsbR1*	[2]
EGD-e *mEos3.2::rsbR1*	This study
EGD-e *ΔrsbL*	[3]
EGD-e *mEos3.2::rsbL*	This study
EGD-e *ΔsigB*	[4]
EGD-e *Δprli42*	[5]
Plasmid	
pMAD	[6]
pMAD mEos3.2-RsbR1	This study
pMAD mEos3.2-RsbL	This study
pNF GFP-RsbR1	This study
pNF mEos3.2	This study
pNF mEos3.2-Prli42	This study
pNF mEos3.2-RsbR1 (including in-frame RsbS and RsbT))	This study
pNF mEos3.2-RsbL	This study
pNF RsbL	This study

Table 3. List of primers

No.	Name	Function	Sequence (5’ □ 3’)	Reference
**1.**	Fw Up_RsbL	Forward primer (carries NcoI site) and reverse primer (carries SalI site) to amplify the 811 bp sequence upstream of the gene *rsbL* (*lmo0799*)	CCACCATGGCAGGATCTGG AAAAAGATC	This study
**2.**	Rv Up_RsbL		GTCGTCGACCTGCGTGTTTC TCCCCCT	This study
**3.**	Fw mEos3.2 (L)	Forward primer (carries SalI site) and reverse primer (carries EcoRI site) to amplify the gene *mEos3.2* with a 6-amino acid linker	GTCGTCGACATGGGAAGTG CGATTAAGCC	This study
**4.**	Rv mEos3.2 (L)		GAAGAATTCCGAACCGCCG GTACCGCCTCGTCTGGCATT GTCAGG	This study
**5.**	Fw RsbL	Forward primer (carries EcoRI site) and reverse primer (carries NcoI site) to amplify the gene *rsbL (lmo0799)* and its 811 bp sequence downstream	GAAGAATTCACCGCTTATCC ACAATTCGATG	This study
**6.**	Rv RsbL		CCACCATGGTTCTGATTCTC CGTAAGCTTTTACC	This study
**7.**	Fw Up_RsbR1	Forward primer (carries NcoI site) and reverse primer (carries SalI site) to amplify the 964 bp sequence upstream of the gene *rsbR1(lmo0889)*	CCACCATGGGCGAAATAAC TCTTTTCTTCAAGGC	This study
**8.**	Rv Up_RsbR1		GTCGTCGACCAGTTATTTTC CAACCTTTCTCCAC	This study
**9.**	Rv mEos3.2 (1)	Reverse primer (carries BamHI site) to amplify the gene *mEos3.2* with 6-amino acid linker; used with the forward primer of Fw mEos3.2 (L)	GGAGGATCCCGAACCGCCG GTACCGCCTCGTCTGGCATT GTCAGG	This study
**10.**	Fw RsbR1	Forward primer (carries BamHI site) and reverse primer (carries NcoI site) to amplify the gene *rsbR1 (lmo0889*) and 1160 bp sequence downstream, including the genes *rsbS*, *rsbT,* and part of *rsbU*	GGAGGATCCTATAAAGATTT TGCAAACTTCATCCG	This study
**11.**	Rv RsbR1_short		CCACCATGGCCCAAAATCA AGTTCATCGTG	This study
**12.**	Fw upup_RsbL	Forward primer at 1126 bp upstream of the gene *rsbL (lmo0799*) to confirm the chromosomal integration	GGTTATTTCCGCAATAGAAA GTAG	This study
**13.**	Rv mid_mEos3.2	Reverse primer in the middle of the gene *mEos3.2* to confirm chromosomal integration	CCTTCCATTGTTATGTCGTTT C	This study
**14.**	Fw mid_mEos3.2	Forward primer in the middle of the gene *mEos3.2* to confirm the chromosomal integration	GGACACTTTCTATAATAAAG TTCGATT	This study
**15.**	Rv downdown_Rsb L	Reverse primer at 1081 bp downstream of gene *rsbL (lmo0799*) to confirm the chromosomal integration	CCAGAACACTTGTTTAATCG C	This study
**16.**	Fw upup_RsbR1	Forward primer at 1015 bp upstream of the gene *rsbR1 (lmo0889)* to confirm the chromosomal integration	GGACTTAAGGGAGTATATC AGCT	This study
**17.**	Rv downdown_Rsb R1	Reverse primer at 2029 bp downstream of the gene *rsbR1 (lmo0889)* to confirm the chromosomal integration	GCTGTATAAGCATCGATCTC C	This study
**18.**	Rv mEosRST	Reverse primer to clone RsbRST from pMAD mEos3.2-RsbR1; carries NcoI site	GCCGCCATGGTTACCGAACC CATTTCG	This study
**19.**	Fw pNF	Forward primer to clone the pNF8 backbone creating pNF mEos3.2-RsbRST; carries NcoI site	CCACCATGGCTGCAGGCATG CAAGC	This study
**20.**	Rv pNF	Reverse primer to clone pNF8 backbone creating pNF mEos3.2-RsbRST; carries SalI site	GTCGTCGACTTAATAAACCT CCTTTCGGATCCGTTG	This study
**21.**	Rv mEosRsbL/ RsbL(pNF)	Reverse primer to clone mEos3.2-RsbRL or rsbL from pMAD mEos3.2-RsbL; carries NcoI site	CCACCATGGTTAGTAAAATT GTAATGCTTCTTTGATTGTG C	This study
**22.**	Fw RsbRL(pNF)	Forward primer to clone *rsbL* (*lmo0799*) from pMAD mEos3.2-RsbL; carries SalI site	GTCGTCGACATGACCGCTTA TCCACAATTCG	This study
**23.**	Fw mEos3.2 pNF8	Forward primer to clone gene for mEos3.2 from pMAD mEos3.2-RsbR1	AGGTTTATTAAAUGGGAAG TGCGATTAAGC	This study
**24.**	Rv mEos3.2 pNF8	Reverse primer to clone gene for mEos3.2 from pMAD mEos3.2-RsbR1	AGTTATTAUGAACCGCCGGT ACCG	This study

**25.**	Fw pNF8 (prli42)	Forward primer to open pNF8 backbone	ATAATAACUGCAGGCATGCAAGCTTG	This study
**26.**	Rv pNF8 mEos3.2	Reverse primer to open pNF8 backbone	ATTTAATAAACCUCCTTTCGGATCCGTTG	This study
**27.**	Fw-pNF8 sequencing	Forward primer for sequencing to check for proper insertion of mEos3.2	TGAGCGCAACGCAATTAATG	This study
**28.**	Rv-pNF8 sequencing	Reverse primer for sequencing to check for proper insertion of mEos3.2	CGATTAAGTTGGGTAACGCCAG	This study
**29.**	Fw prli42 (res)	Forward primer to amplifying *prli42* from pAD prli42; carries NcoI site	CCACCATGGATGACTAATAAAAAAGTAGTTCGC	This study
**30.**	Rv prli42 (res)	Reverse primer to amplifying *prli42* from pAD prli42; carries SalI site	GTCGTCGACTAAAAACATTAATACCCCGGTT	This study
**31.**	Fw pNFmEos (res)	Forward primer to amplifying pNF mEos3.2; carries SalI site	GTCGTCGACTAATAACTGCAGGCATGCAAGC	This study
**32.**	Rv pNFmEos (res)	Reverse primer to amplifying pNF mEos3.2; carries NcoI site	CCACCATGGTGAACCGCCGGTACCG	This study

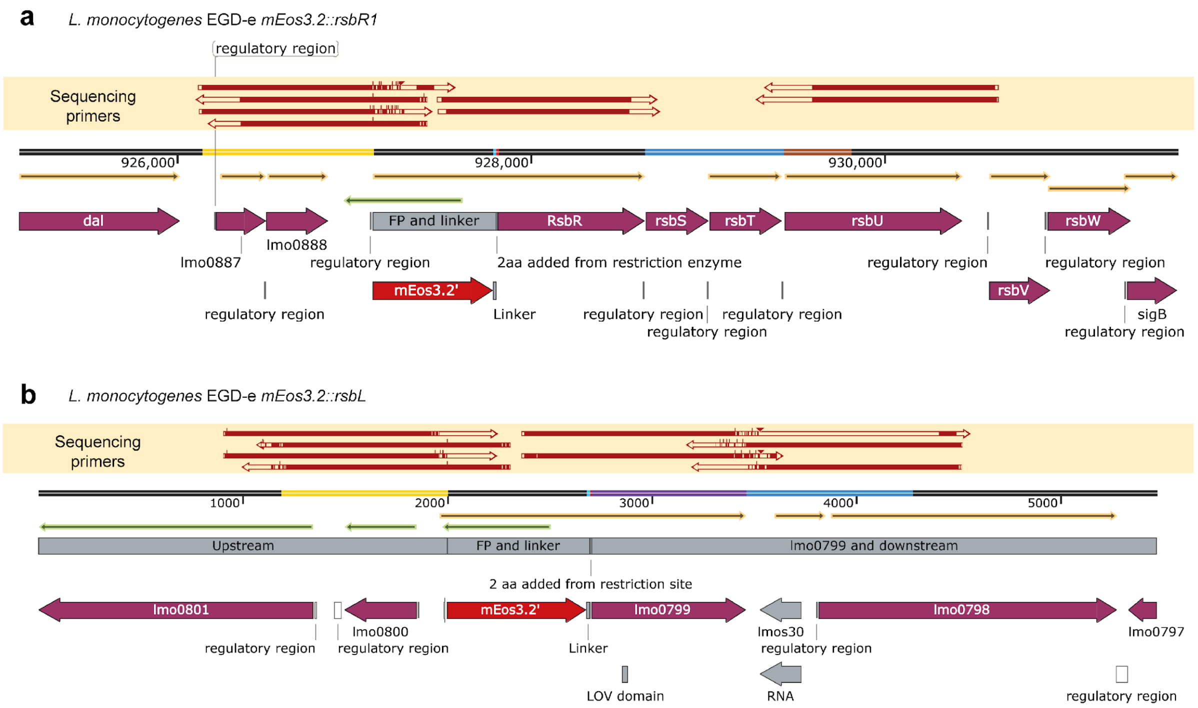

Supplementary Fig. 1 | Sequencing results of the integration strains. **a**, and **b**, *L. monocytogenes* EGD-e *mEos3.2::rsbR1* and *L. monocytogenes* EGD-e *mEos3.2::rsbL*, respectively. Two colonies of each integrant were sequenced. After chromosomal integration of *mEos3.2::rsbR1* and *mEos3.2::rsbL*, the plasmids were excised as shown by X-gal (blue-white) and antibiotic sensitivity screens. Selected colonies were checked for integration by colony-PCR and sequencing with primers upstream, downstream, and two middle positions of the gene *mEos3.2*.

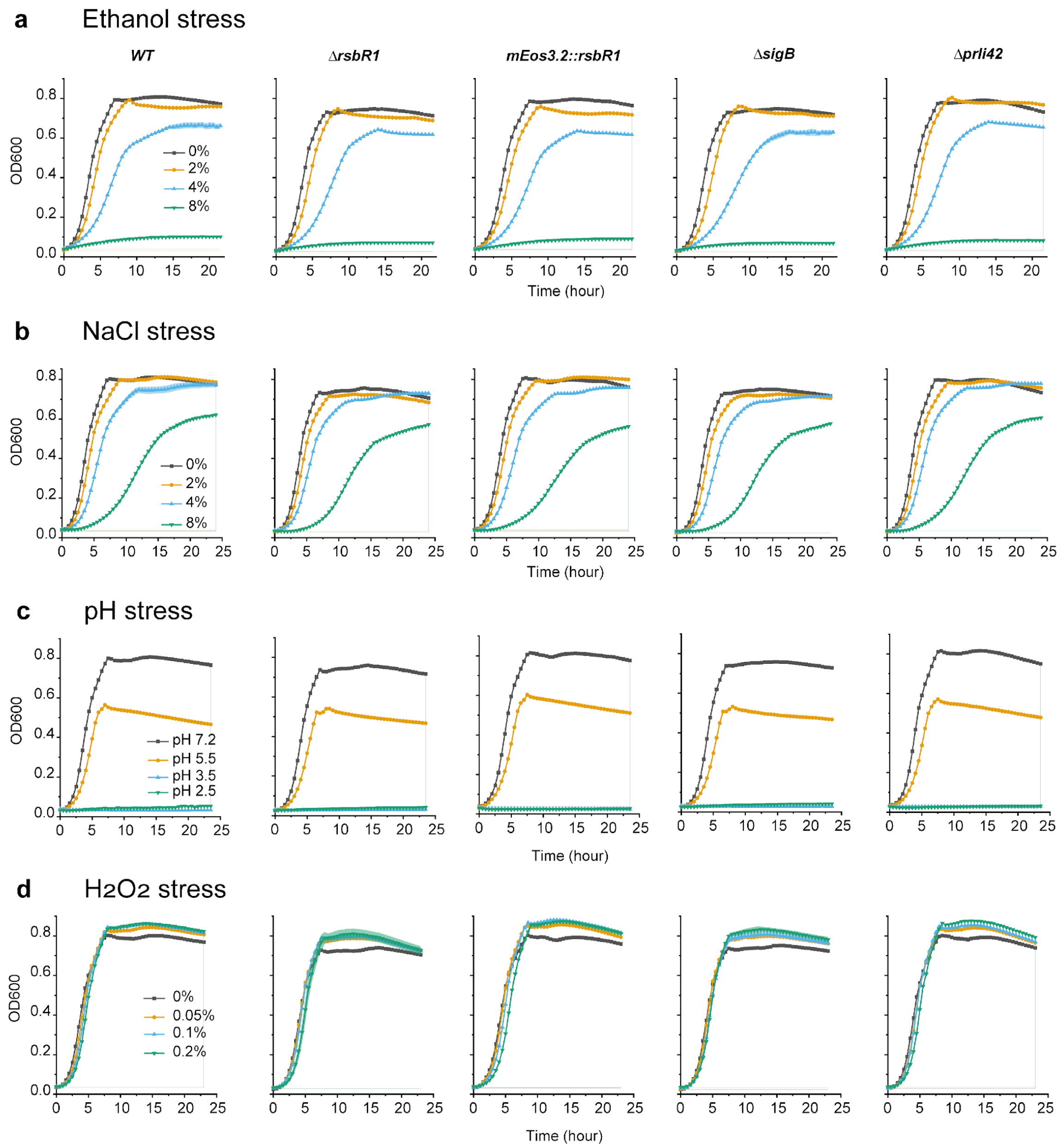

Supplementary Fig. 2 | Growth under ethanol, NaCl, low pH, and H_2O_2 stress of *L. monocytogenes* wild-type, *ΔrsbR1,* integrant *mEos3.2::rsbR1, ΔsigB,* and *Δprli42*. The growth was monitored in 96-well plates at 30 °C with 200 rpm shaking. Cells were grown in BHI medium supplemented with **(a)** ethanol at 0% (control), 2%, 4%, and 8% ($v.v^{-1}$); **(b)** NaCl at 0% (control), 2%, 4%, and 8% ($w.v^{-1}$); **(c)** HCl to set the pH to pH 7.2 (control), pH 5.5, pH 3.5, and pH 2.5; and **(d)** with H_2O_2 at 0% (control), 0.05%, 0.1% and 0.2%.

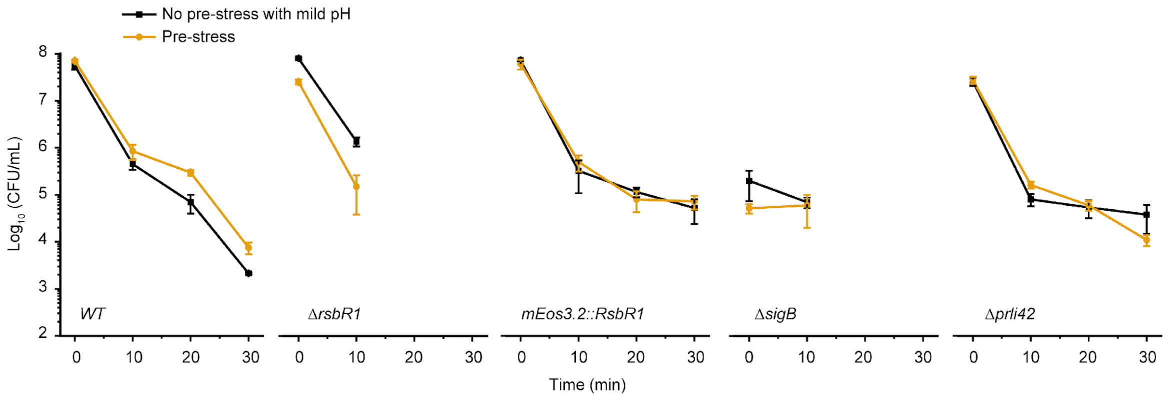

Supplementary Fig. 3 | Phenotypic test of survival of *L. monocytogenes* upon acid shock treatment. Mid-log phase cultures untreated at pH 7.2 (No pre-stress) or treated at pH 5.0 (Pre-stress) for 15 min and subsequently challenged in acidified BHI (pH 2.5). The detection threshold is 10^2 CFU.mL^{-1}. Samples were taken at 0, 10, 20, and 30 min. Survival data are expressed as Log_{10} (CFU.mL^{-1}). *ΔrsbR1* and *ΔsigB* were notably more sensitive to the acid treatment than wild-type, *mEos3.2::rsbR1*, and *Δprli42* and colonies were no longer detected after 10 min treatment at pH 2.5. Error bars represent standard deviations. Three biological replicates were made.

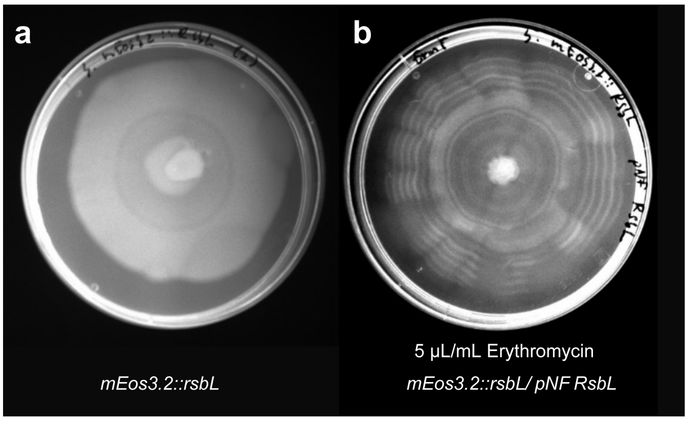

Supplementary Fig. 4 | Ring formation phenotype on low-agar after twelve oscillating cycles of light and dark (12-hour intervals). a, and **b,** The integration strain *mEos3.2::rsbRL* and the same strain expressing *rsbL* from the P_{dlt} promoter of pNF RsbL. We used an erythromycin concentration for plasmid selection of 5 µg.mL^{-1}. Images are representative of at least three independent experiments.

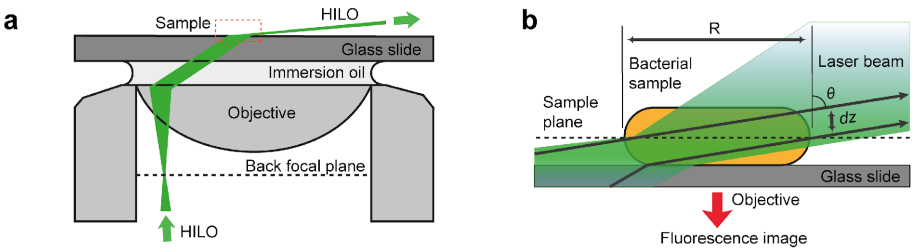

Supplementary Fig. 5 | Schematic of the highly inclined and laminated optical sheet (HILO) microscopy, adapted from the previous study[7]. **a**, The set-up of optics. **b**, Bacterial sample is illuminated with a laminated thin sheet of the laser beam. The *dz* is the thickness of the geometrical optics along the z-direction and $dz = R/\tan\theta$, where *R* is the diameter of the illuminated area, and θ is the incidence angle at the sample.

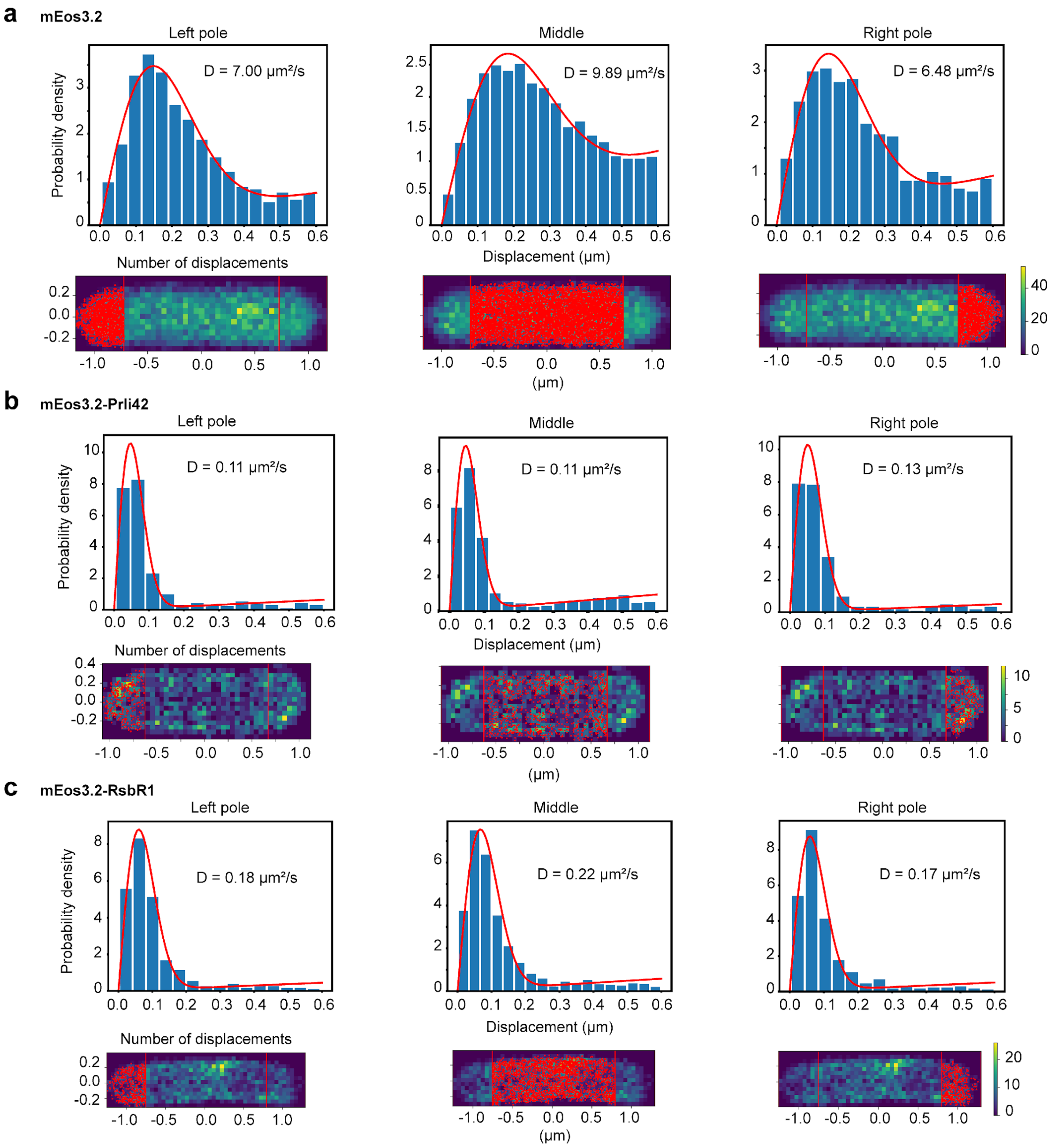

Supplementary Fig. 6 | Diffusion at subcellular regions. a, **b**, and **c**, show the subcellular division of free mEos3.2, mEos3.2-Prli42, and mEos3.2-RsbR1, respectively. Cells were divided into three main regions: the cell center (60% in length) and the cell poles (each 20% in length). The displacements belonging to the corresponding regions were then analyzed separately. The top panels are the distributions of all single-molecule displacements from the corresponding regions shown in the bottom panels. The red curve is obtained from MLE fitting, using equation (4), and the resulting diffusion coefficients are presented inside the panels.

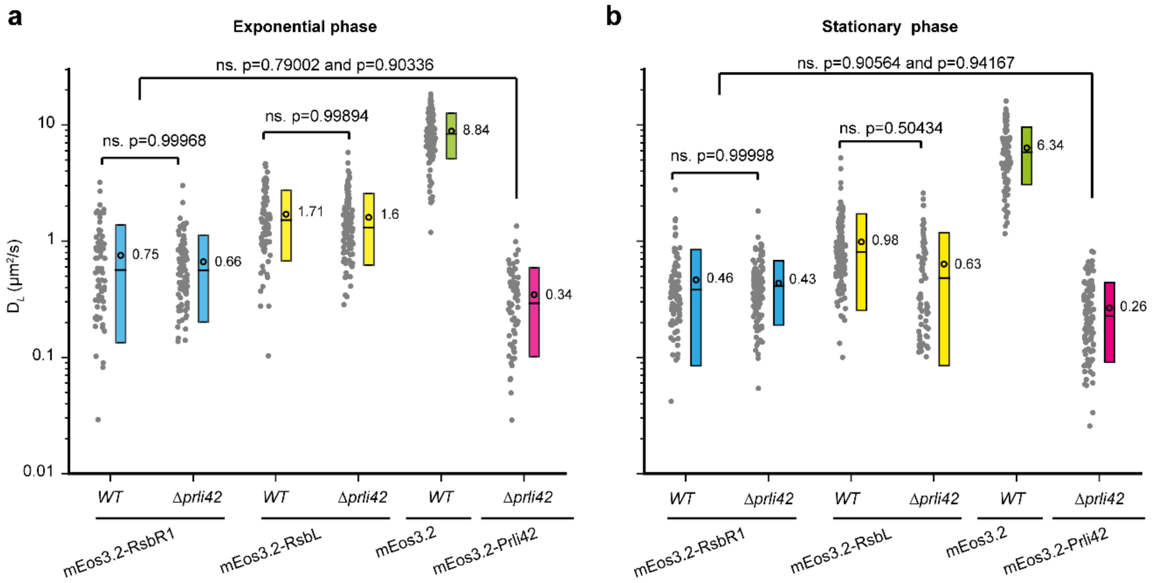

Supplementary Fig. 7 | Ensemble diffusion coefficients determined by FRAP measurements. **a,** and **b,** Box charts of the D_L from FRAP measurements showing the ensemble mobility of mEos3.2-RsbR1, mEos3.2-RsbL, mEos3.2-Prli42, and mEos3.2 in the wild-type and *Δprli42* strains in the exponential and stationary phase of growth. Each dot shows the data of one cell. The box range indicates the standard deviation (SD), and the open circle and dashed symbols inside the boxes indicate the mean and median, respectively. (ns) is not significant and statistical significance was determined by one-way ANOVA followed by Tukey's post-hoc test to calculate P-values.

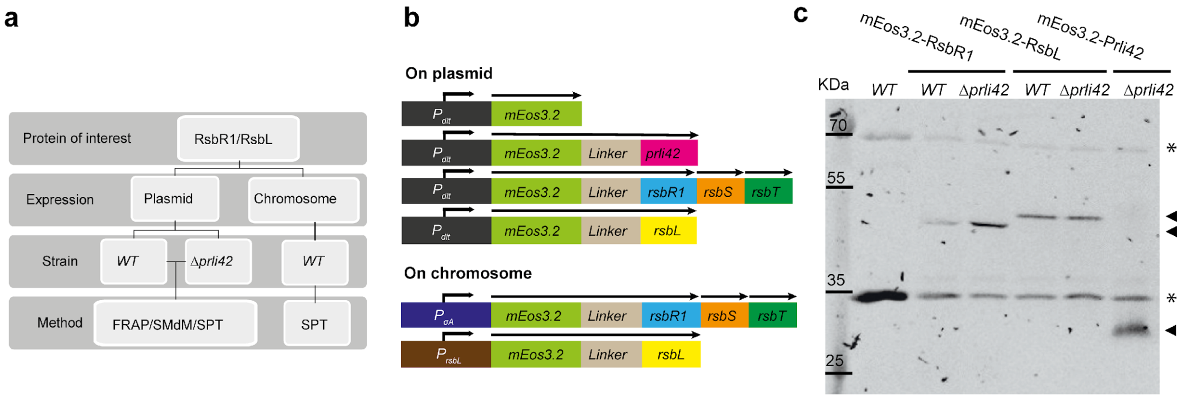

Supplementary Fig. 8 | Experimental overview, protein tagging strategy, electrophoretic mobility, and in-gel fluorescence of proteins. a, Experimental overview for measuring the intracellular dynamics of stressosome proteins in live cells. The targeted proteins are RsbR1, and RsbL produced either from a multi-copy plasmid or the chromosome in wild-type (WT) or *Δprli42* strains. The diffusion coefficients of the proteins are measured by FRAP, SMdM, or SPT. **b**, A photoconvertible fluorescent protein mEos3.2 is fused to the N-terminus of mEos3.2-RsbR1 and mEos3.2-RsbL, and the fusion proteins are produced from a plasmid or the chromosome. Free cytosolic mEos3.2 and mEos3.2 fused to the N-terminus of Prli42 (mEos3.2-Prli42) are produced from pNF mEos3.2-Prli42, which is under the control of the constitutive P_{dlt} promoter; while the chromosomal expression is driven by corresponding native promoters, which are P_{sigA} and P_{rsbL} for *rsbR1* and *rsbL*, respectively. **c**, In-gel fluorescence of the constructs tagged with mEos3.2 produced in the wild-type and *Δprli42* strains. The expected bands are shown by black triangle symbols. A star symbol represents endogenous fluorescence in *L. monocytogenes*. The molecular weight (kDa) of the marker proteins is indicated on the left of the panel. These results indicate that the tandem fusions inside *L. monocytogenes* are intact.

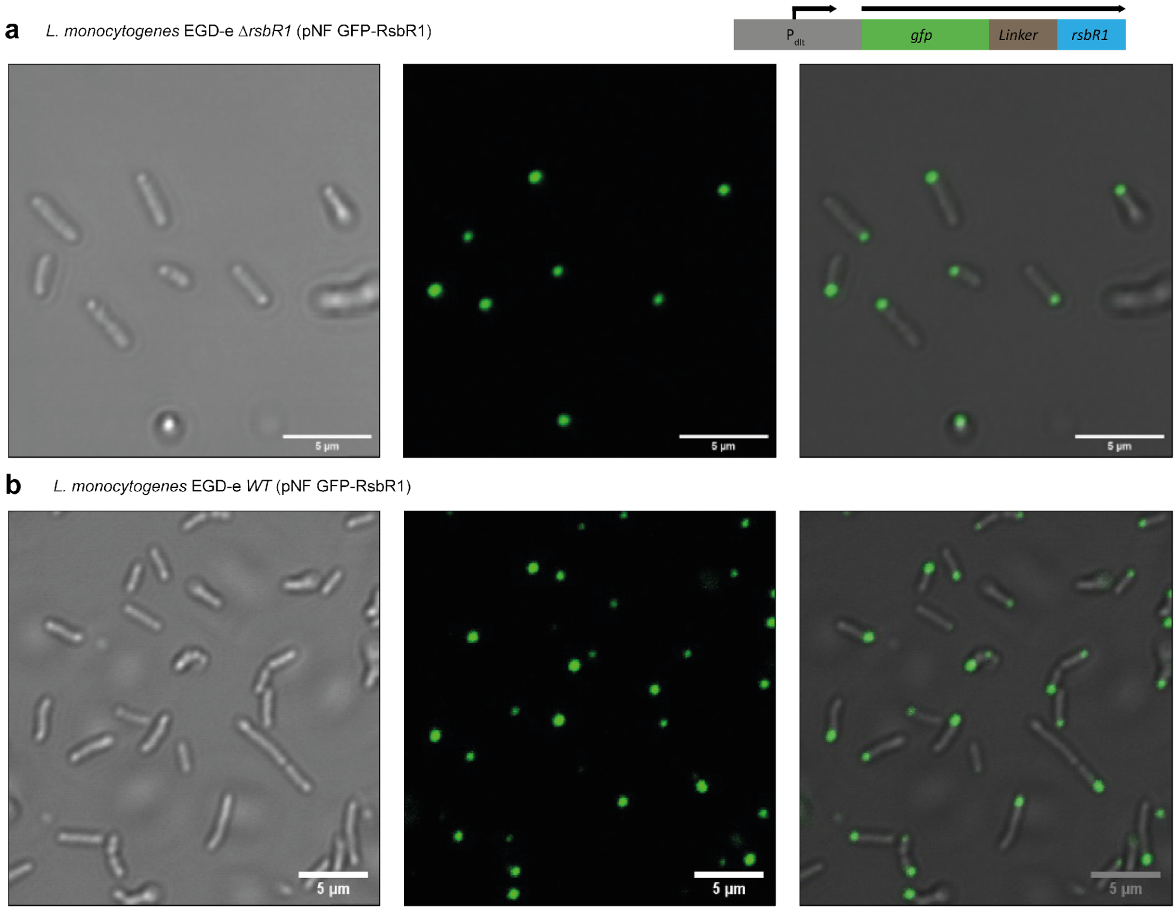

Supplementary Fig. 9 | Pole aggregation of GFP-RsbR1 observed by confocal microscopy. The green fluorescent protein GFP was fused to the N-terminal end of RsbR1 on pNF GFP-RsbR1 under the control of the P_{dlt} promoter. **a**, and **b**, The expression of *gfp-rsbR1* in *ΔrsbR1* and wild-type strains is shown, respectively. Left panels are the bright field; middle panels are the fluorescence upon 488 nm excitation; right panels are the overlays of the left and middle panels. Scale bars are 5 µm.

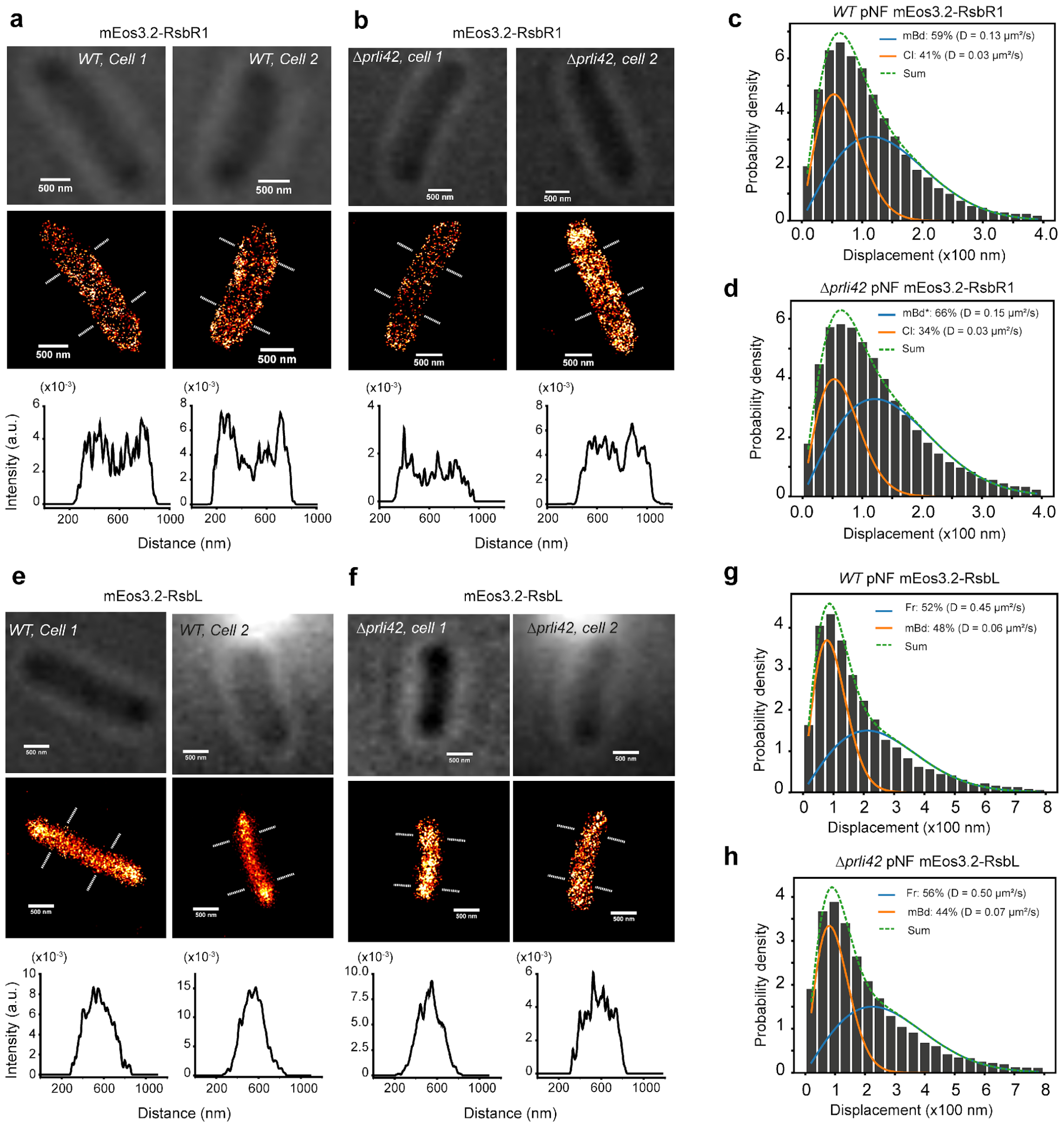

Supplementary Fig. 10 | Localization by PALM and lateral diffusion by SPT of mEos3.2-RsbR1 and mEos3.2-RsbL. **a**, and **b**, Localization of mEos3.2-RsbR1 in wild-type and *Δprli42* strains, respectively. The top panels are bright-field images. Middle panels are reconstructed images from a series of frames. All fusion proteins were expressed from the P_{dlt} promoter of pNF mEos3.2-RsbR1, and pNF mEos3.2-RsbL Scale bars are 500 nm. **c**, and **d**, Distributions of single-molecule displacements of mEos3.2-RsbR1 in wild-type and *Δprli42* strains, respectively. Blue and orange curves, showing membrane-bound (mBd) and (Cl) fractions, were obtained from fitting the histogram to the two-component 2D random walk model using equation (6). The starred membrane-bound fraction (mBd*) indicates mEos3.2-RsbR1 molecules loosely associated with the membrane in the *Δprli42* strain. The green

dashed line is the sum of two fractions. **e**, and **f**, Localization of mEos3.2-RsbL in wild-type and *Δprli42* strains, respectively. Similar to **(c)** and **(d)**, **g**, and **h**, are distributions of single-molecule displacements of mEos3.2-RsbL. Differences in maximum displacement distances (r_{max}) were chosen based on the diffusion constants of the proteins.

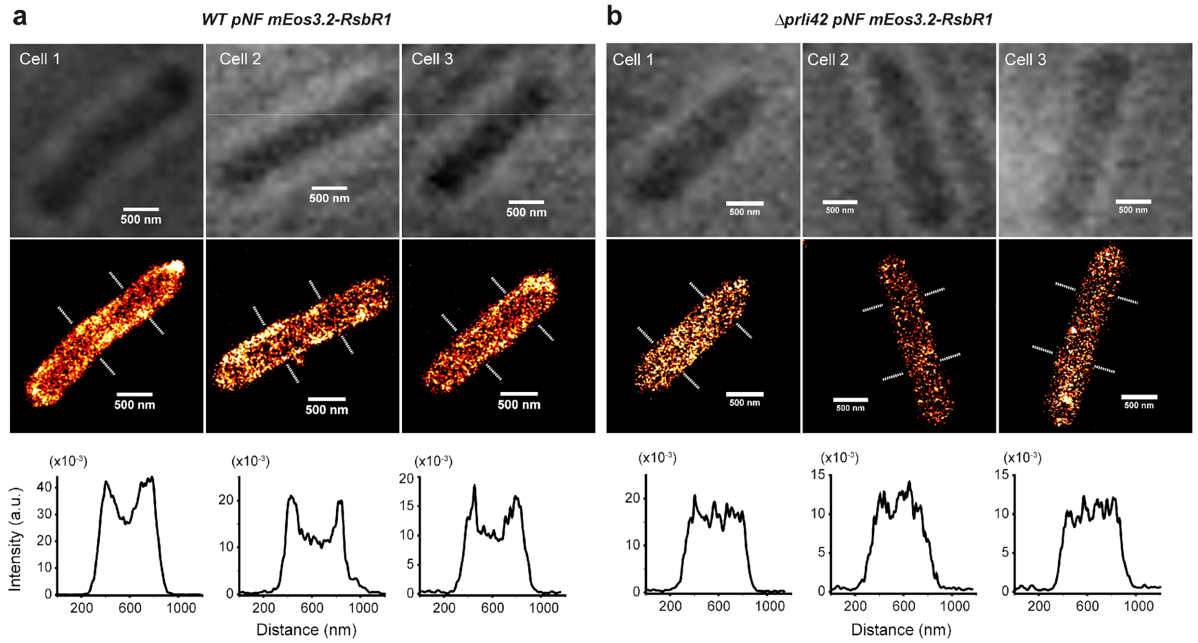

Supplementary Fig. 11 | Reconstruction images of mEos3.2-RsbR1. The tandem fusion was synthesized in cells from the P_{dlt} promoter of pNF mEos3.2-RsbR1 and performing SMdM measurements (65,000 frames). Single-molecule detection was done using equation (5). **a**, Membrane localizations are detected in the wild-type strain. **b**, Most localized molecules are found in the cytoplasm in the *Δprli42* strain.

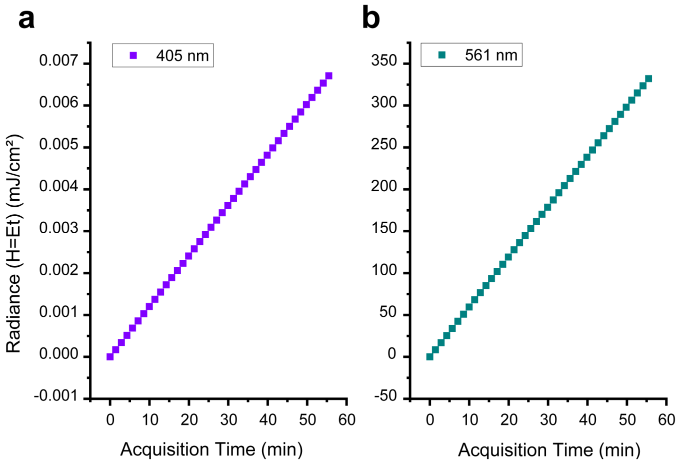

Supplementary Fig. 12 | Radiance graph (H = E·t). H is the radiant exposure dose in $mJ.cm^{-2}$, E is the irradiance in $mW.cm^{-2}$, and t is the exposure time in seconds. The acquisition frequency is 56 Hz; the pulse time of **(a)** 405 nm and **(b)** 561 nm are 1 ms and 0.5 ms, respectively; 405 nm pulses in every 2 frames, and 561 nm pulses in every frame; hence, in 1 second, the radiance time of 405 nm and 561 ms are 28 ms.

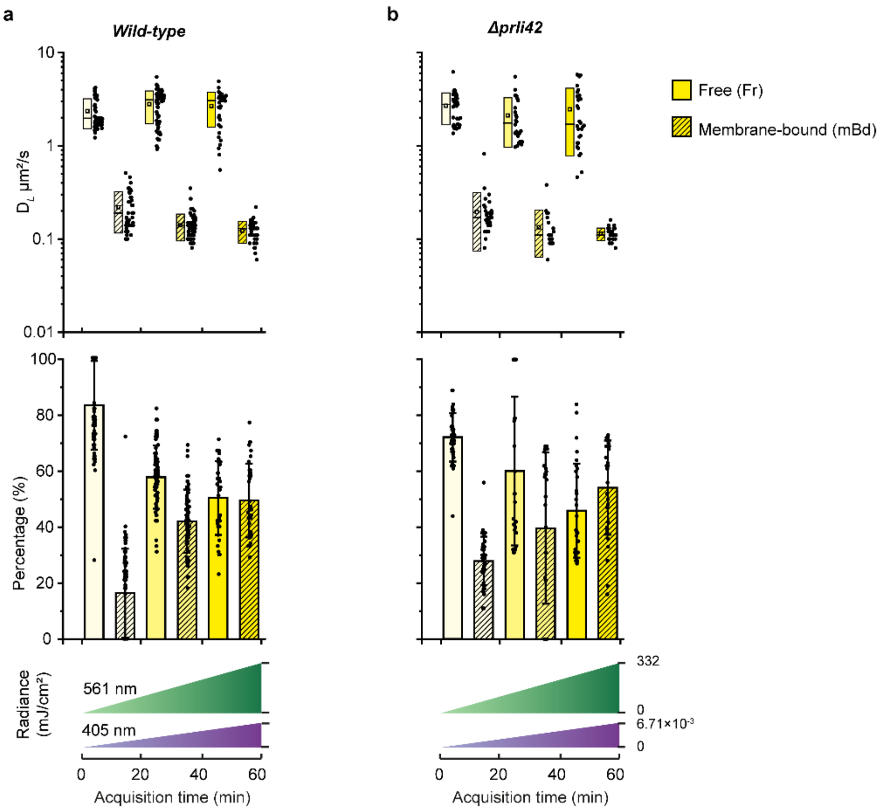

Supplementary Fig. 13 | RsbL clustering upon laser light irradiation is not Prli42-dependent. a, and **b**, Lateral diffusion of mEos3.2-RsbL in 3 continuous SMdM acquisitions (each of ~20 min) in wild-type and *Δprli42* strains, respectively. Top panel: box charts of the D_L with the free (Fr) fraction and membrane-bound (mBd) fractions. Each dot shows the data of one cell. Box range indicates the standard deviation (SD), and open square and dash symbols inside the boxes indicate the mean and median, respectively. Middle panel: the corresponding percentage of the fractions is in the top panel. Error bars represent standard deviations. Bottom panel: the radiance of 561 nm and 405 nm lasers were measured at the focus above the glass slide during the acquisition.

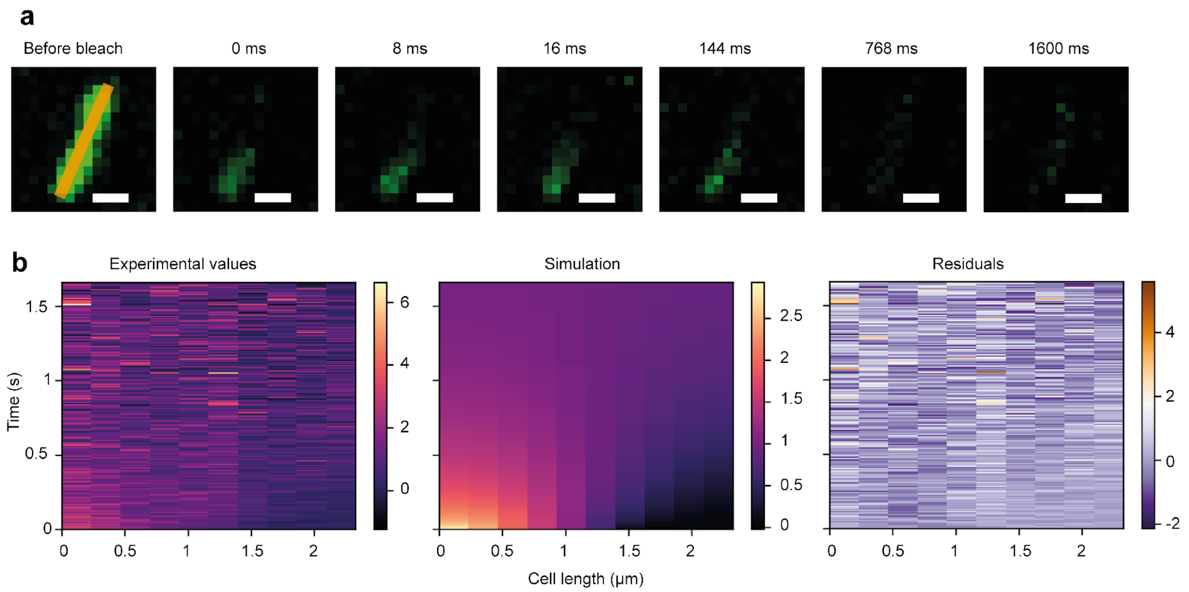

Supplementary Fig. 14 | FRAP acquisition to determine ensemble diffusion coefficients. **a**, An example of a FRAP acquisition and the fluorescence recovery for the diffusion constant of 1.03±0.06 μm^2.s^{-1}. The orange line marks the analyzed region; each acquisition has 200 cycles, and the average intensity before bleaching (the first 3 cycles) is used to normalize the fluorescence. Time zero was recorded immediately after the photobleaching. The scale bar is 1 μm. **b**, The fluorescent intensity along the orange line of the labelled cell of the panel **(a)** in time for the experimental data (left), the one-dimensional heat-equation simulation (middle), and the residuals (right).

References

1. Ho, F. Y. & Poolman, B. *Engineering escherichia coli for functional expression of membrane proteins*. *Methods in Enzymology* **556**, (Elsevier Inc., 2015).
2. O'Donoghue, B. A molecular genetic investigation into stress sensing in the food-borne pathogen Listeria Monocytogenes: roles for RsbR and its paralogues. (2016).
3. O'Donoghue, B. *et al.* Blue-light inhibition of Listeria monocytogenes growth is mediated by reactive oxygen species and is Influenced by σB and the blue-light sensor Lmo0799. *Appl. Environ. Microbiol.* **82**, 4017–4027 (2016).
4. Guerreiro, D. N. *et al.* Mild Stress Conditions during Laboratory Culture Promote the Proliferation of Mutations That Negatively Affect Sigma B Activity in Listeria monocytogenes. *J. Bacteriol.* **202**, 1–20 (2020).
5. Impens, F. *et al.* N-terminomics identifies Prli42 as a membrane miniprotein conserved in Firmicutes and critical for stressosome activation in Listeria monocytogenes. *Nat. Microbiol.* **2**, (2017).
6. Arnaud, M., Chastanet, A. & De, M. New vector for efficient allelic replacement in naturally nontransformable, low-GC-content, Gram-positive bacteria. *Appl. Enviromental Microbiol.* **70**, 6887–6891 (2004).
7. Tokunaga, M., Imamoto, N. & Sakata-Sogawa, K. Highly inclined thin illumination enables clear single-molecule imaging in cells. *Nat. Methods* **5**, 159–161 (2008).
