# Supplementary material for: Super-resolving microscopy reveals the localizations and movement dynamics of stressosome proteins in Listeria monocytogenes
Source: Commun Biol. 2023 Jan 14;6:51. doi: 10.1038/s42003-023-04423-y (PMC9840623; doi:10.1038/s42003-023-04423-y)
Supplement: Supplementary file 3 — Description of Additional Supplementary Files [file 42003_2023_4423_MOESM3_ESM.pdf]

Description of Additional Supplementary Files

File name: Supplementary Video 1
Description: Localization of mEos3.2-RsbR1 in L. monocytogenes

File name: Supplementary Video 2
Description: Clustering of mEos3.2-RsbR1 in L. monocytogenes (overlaid with a phase contrast image)

File name: Supplementary Video 3
Description: Clustering upon irradiation of mEos3.2-RsbL in L. monocytogenes

File name: Supplementary Data 1
Description: Excel file with all diffusion coefficients reported in the paper
